# Renal Disease Simulating Calculus in the Bladder

**Published:** 1843-06

**Authors:** 


*Renal disease Simulating Calculus in the Bladder.*—Segalas reports that he was called to attend on a child, from four to five years of age, which often placed its hands on the genitals, and experienced frequent desire to pass urine, which action, however, was always performed with pain. The urine was tolerably abundant, and yielded a muco-purulent deposit. The acuteness of the various symptoms had been considerably increased during some recent attacks of roseola and bronchitis. These symptoms apparently indicated the presence of a calculus in the bladder; Segalas, however, doubted that one really existed, having never met with this disease in any children but those of parents in a much lower sphere of life than those of this patient; and a careful examination proved that no urinary calculus in reality existed. Some time afterwards the child died of a cerebral affection, and the body was opened. The bladder, as well as the urethra, was found perfectly healthy and free from urinary deposit; but the left ureter was much dilated, and the pelvis of the corresponding kidney three or four times larger than that of the opposite side. The calices also were enlarged, and the cortical substance of the kidney was inflamed at several points. The right kidney was healthy.—*Lond. Lancet*, from *Gaz. des Hôp.*, Jan. 7.

